# Advancements in Surgical Approaches for Sacrococcygeal Pilonidal Sinus: A Comprehensive Review

**DOI:** 10.7759/cureus.68502

**Published:** 2024-09-03

**Authors:** Vishal S Shinde, Suhas Jajoo, Raju K Shinde

**Affiliations:** 1 Surgery, Jawaharlal Nehru Medical College, Datta Meghe Institute of Higher Education and Research, Wardha, IND; 2 General Surgery, Jawaharlal Nehru Medical College, Datta Meghe Institute of Higher Education and Research, Wardha, IND

**Keywords:** advanced technologies, recurrence rates, flap-based repairs, minimally invasive techniques, surgical approaches, sacrococcygeal pilonidal sinus

## Abstract

Sacrococcygeal pilonidal sinus (SPS) is a condition involving the formation of a cavity in the lower back region. It is more common among young adults and is influenced by factors such as sitting for long periods, body hair, and certain lifestyle habits. Surgical treatment is often necessary for recurring or severe cases, and various surgical techniques available, ranging from traditional surgical methods to newer, less invasive approaches. This comprehensive review examines the progress in surgical techniques for managing SPS, emphasizing the effectiveness, safety, and patient outcomes associated with different methods. It provides an overview of traditional procedures, such as excision with primary closure, and contrasts these with recent innovations like endoscopic and laser-assisted techniques. The review also considers advanced technologies, including the potential of robotic surgery and the use of specialized materials. By assessing clinical outcomes, recurrence rates, complications, and patient satisfaction, this review seeks to identify the most effective surgical strategies for SPS. Additionally, it discusses recent technological advancements and highlights areas needing further research to improve the management and treatment of this condition.

## Introduction and background

Sacrococcygeal pilonidal sinus (SPS) is a chronic inflammatory condition characterized by the presence of a sinus or cavity near the sacrococcygeal region, often containing hair and debris [1]. This condition typically manifests as a painful, swollen area at the base of the spine, which can become infected and form abscesses. SPS is most commonly seen in young adults, particularly males, though it can affect individuals of any age and gender [2]. The etiology of SPS is often attributed to the entrapment of hair follicles, leading to inflammation and the formation of a sinus tract. The condition is notably prevalent among individuals with sedentary lifestyles, familial predispositions, and excessive body hair, and is more common in occupations involving prolonged sitting, such as truck drivers or office workers [3].

The importance of reviewing surgical approaches for sacrococcygeal pilonidal sinus stems from the condition’s impact on quality of life and the variety of surgical techniques available for its treatment [4]. While conservative management can be effective in some cases, surgical intervention is often necessary for recurrent or severe cases. Over the years, various surgical methods have been developed and refined, including traditional excision techniques, flap-based repairs, and more recent minimally invasive approaches. Each technique varies regarding recurrence rates, postoperative pain, recovery time, and overall patient outcomes. Reviewing these approaches is essential for updating clinical practice and providing evidence-based recommendations [5].

This review aims to provide a comprehensive overview of traditional and contemporary surgical techniques for managing sacrococcygeal pilonidal sinus, assess their clinical outcomes, and explore recent advancements in surgical methods and technologies. By evaluating the effectiveness, safety, and impact of different surgical strategies, this review aims to enhance the understanding of optimal treatment options and guide clinicians in selecting the most appropriate approach for their patients. Additionally, this review seeks to identify gaps in current research and suggest areas for further investigation to improve the management and outcomes of sacrococcygeal pilonidal sinus.

## Review

Pathophysiology and classification

**Figure 1:**
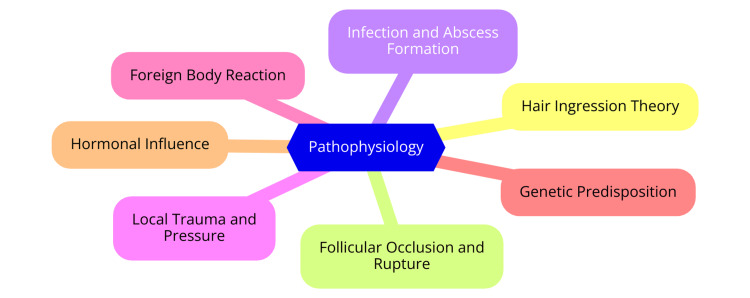
Pathophysiology of sacrococcygeal pilonidal sinus Image Credit: Dr Vishal S. Shinde

Pilonidal sinus disease is a prevalent condition characterized by forming a small hole or tunnel in the skin, typically located in the natal cleft at the top of the buttocks. Understanding its pathophysiology, classification, and associated risk factors is essential for effective management and treatment [6]. Historically, pilonidal sinus disease was believed to be congenital; however, current understanding indicates that it is primarily an acquired condition. The term "pilonidal" is derived from the Latin words *pilus* (hair) and *nidus* (nest), reflecting its association with hair follicles. The condition usually arises when loose hairs penetrate the skin, leading to inflammation and infection. This process is exacerbated by friction, prolonged sitting, and other factors that force hair into the skin, triggering an immune response that forms a cyst around the hair [7]. The pathophysiology involves accumulating hair, debris, and pus within the sinus, which can become infected. Inflammation may lead to abscess formation, and recurrent infections can result in chronic symptoms or complications, such as the rare development of squamous cell carcinoma [8]. Although there is no universally accepted classification system for pilonidal sinus disease, it is often categorized based on clinical presentation. Acute pilonidal sinus is marked by sudden onset of symptoms, including pain and swelling, often due to infection or abscess formation. In contrast, chronic pilonidal sinus is characterized by persistent or recurrent symptoms, with the sinus intermittently discharging pus or hair without acute inflammation. Some clinicians may use staging systems based on disease extent and complications, though these systems vary widely [9]. Pilonidal sinus disease predominantly affects young adults, particularly males, with a male-to-female ratio ranging from 2.2:1 to 4:1. The condition is most common in individuals aged 15-30 years, with a significant decrease in incidence after age 40. Several factors increase the risk of developing pilonidal sinus, including obesity, which can contribute to a deeper natal cleft and increased friction [10]. A sedentary lifestyle, especially occupations involving prolonged sitting (e.g., truck drivers and office workers), is associated with higher incidence rates. Hair characteristics also play a significant role; individuals with coarse or curly hair are more prone to hair penetration into the skin, contributing to sinus formation. Family history may indicate a genetic predisposition, as the condition can run in families. Poor hygiene practices can also heighten the risk of infection, further complicating the condition [11]. The pathophysiology is illustrated in Figure 1.

Traditional surgical approaches

Excision and Primary Closure

Excision with primary closure involves surgically removing the pilonidal sinus and immediately closing the wound. This procedure can be performed through midline or lateral incisions to eliminate the sinus and promote rapid healing by suturing the wound edges together [12]. Although this method facilitates a swift recovery, it is associated with a higher recurrence rate, which has been reported to be between 10% and 30% in various studies. Potential complications include wound infection, seroma formation, and delayed healing. The tension on the wound edges during closure can exacerbate these issues, resulting in a higher risk of recurrence compared to alternative techniques [13].

Excision with Open Wound Healing

In contrast, excision with open wound healing involves surgically removing the pilonidal sinus and allowing the wound to heal by secondary intention. This means the wound is left open and heals naturally from the inside out. The wound is regularly packed and dressed to facilitate drainage and prevent infection [14]. This approach is especially beneficial when there is a risk of tension on the wound edges that could hinder healing. Healing by secondary intention generally results in a lower recurrence rate, estimated to be between 8% and 21%. However, this technique necessitates extensive postoperative care, including frequent dressing changes and vigilant monitoring for infection. The healing process can take several weeks to months, which may be inconvenient for patients. Despite the longer healing time, the resulting broader and flatter scar can help reduce friction and hair penetration, thus decreasing the likelihood of recurrence [15].

Flap-Based Techniques

Flap-based techniques, such as the Limberg and Karydakis flap, have become increasingly popular due to their effectiveness in reducing recurrence rates. The Limberg flap technique involves excising the pilonidal sinus and reconstructing the defect with a rhomboid-shaped skin flap. This method facilitates tension-free closure and has shown promising results in minimizing recurrence. Similarly, the Karydakis flap technique uses an asymmetrical incision and transposed flap to cover the defect. Like the Limberg flap, it aims to reduce tension and enhance cosmetic outcomes [16]. Both flap techniques have been associated with lower recurrence rates than primary closure, often reported as below 10%. While complications such as flap necrosis, infection, and delayed healing can occur, they are generally less frequent than those occurring when traditional methods are employed. Aesthetic outcomes are usually favourable, with patients frequently reporting higher satisfaction due to improved cosmetic appearance and reduced recurrence [17]. Traditional surgical approaches for sacrococcygeal pilonidal sinus are detailed in Table 1.

**Table 1:** Traditional Surgical Approaches for Sacrococcygeal Pilonidal Sinus (SPS)

Surgical Approach	Description	Advantages	Disadvantages
Excision with Primary Closure	Removal of the sinus tract followed by direct suturing of the wound edges.	Faster healing time, less wound care required.	Higher recurrence rate, risk of wound dehiscence, potential infection.
Wide Excision with Secondary Healing	Complete excision of sinus and the surrounding tissue, allowing the wound to heal by granulation (without suturing).	Lower recurrence rate, effective for complex/recurrent cases.	Prolonged wound healing time and intensive wound care are needed, and there is potential for discomfort and infection.
Bascom Procedure	Minimal excision to remove only the sinus tract and pits, with preservation of surrounding skin and subcutaneous tissue.	Minimally invasive, reduced healing time, less postoperative pain.	Potential for incomplete removal, risk of recurrence if tracts not fully excised.
Karydakis Flap	Lateral incision and excision with a flap of skin and subcutaneous tissue shifted to cover the midline, preventing recurrence.	Low recurrence rates, off-midline closure reduces tension.	Technical complexity, longer operative time, potential for flap necrosis.
Limberg Flap	Rhomboid-shaped excision with a transposition flap from the gluteal region to cover the defect.	Effective for complex/recurrent cases, low recurrence rates.	Requires advanced surgical skills, potential for flap complications.
Z-Plasty	Excision of sinus with Z-shaped incision to redistribute tension and minimize scarring.	Reduced midline tension, less recurrence, aesthetic benefits.	Technically demanding, risk of flap necrosis, potential for more scarring if not properly performed.

Recent advancements in surgical techniques

Recent advancements in surgical techniques have significantly transformed minimally invasive surgery (MIS), leading to improved patient outcomes and reduced recovery times. Among these innovations, minimally invasive methods such as endoscopic and laser-assisted approaches have gained prominence [18]. These techniques involve making small incisions to minimize bodily trauma, allowing for procedures to be performed with enhanced precision. Endoscopic procedures utilize a camera and specialized instruments inserted through small openings. At the same time, laser-assisted techniques use focused light to cut or coagulate tissue, effectively minimising bleeding and promoting faster recovery [19]. The advantages of these minimally invasive approaches are substantial, including reduced postoperative pain, shorter hospital stays, and lower complication rates. Patients often experience quicker recoveries and less scarring than traditional open surgery, making MIS a preferred option for various surgical interventions [20]. In addition to minimally invasive techniques, recent innovations have introduced biological meshes in surgical repairs, particularly in hernia surgeries. These biological meshes are designed to integrate with the body's tissues, enhancing healing while reducing the risk of complications associated with synthetic materials [21]. Their use has been linked to improved patient outcomes and lower recurrence rates, representing a significant advancement in surgical practice. Furthermore, the integration of tissue engineering and regenerative medicine into surgical techniques is gaining traction. Approaches that promote tissue regeneration, including stem cells and growth factors, are being explored to enhance healing and functional recovery after surgery. This innovative direction promises to improve outcomes across various surgical contexts [22]. The application of advanced technologies has also been pivotal in advancing surgical techniques. Robotic-assisted surgery has emerged as a groundbreaking development in minimally invasive procedures. This technique allows surgeons to perform complex operations with enhanced precision and control through robotic arms [23]. The 3D visualisation of robotic systems enables superior manoeuvrability in confined spaces, improving surgical outcomes. Additionally, advanced navigation systems and image-guided surgery technologies have revolutionized the approach to complex procedures. These systems provide real-time imaging and guidance, allowing for more accurate targeting of tissues and structures during surgery. This technology is particularly beneficial in neurosurgery and orthopaedics, where precision is critical [24]. Recent advancements in surgical techniques for sacrococcygeal pilonidal sinus treatment are detailed in Table 2.

**Table 2:** Recent Advancements in Surgical Techniques for Sacrococcygeal Pilonidal Sinus (SPS) Treatment

Technique	Description	Benefits	Limitations	Outcomes
Endoscopic Pilonidal Sinus Treatment (EPSiT)	Minimally invasive procedure using an endoscope to visualize and remove hair and debris from the sinus tract.	Reduced postoperative pain, quicker recovery, minimal scarring, and lower complication rates.	Requires specialized training and equipment; may not be suitable for large or complex sinuses.	Low recurrence rates and high patient satisfaction due to minimally invasive nature.
Laser-Assisted Pilonidal Sinus Treatment (LAPS)	Uses laser energy to ablate the pilonidal sinus tract and seal the wound.	Precise tissue ablation, reduced blood loss, shorter healing time, and decreased risk of infection.	High initial cost of laser equipment; potential for burns or tissue damage if not used correctly.	Effective in reducing recurrence with less postoperative discomfort and faster return to activities.
Robotic-Assisted Surgery	Utilizes robotic systems to perform precise excision and closure of the pilonidal sinus.	Enhanced precision, reduced blood loss, lower complication rates, and quicker recovery.	High cost and limited availability; requires advanced surgical skills and training.	Lower recurrence rates and shorter hospital stays compared to traditional methods.
Flap-Based Techniques (e.g., Limberg, Karydakis)	Surgical reconstruction using local tissue flaps to cover the excised sinus area.	Lower recurrence rates due to tension-free closure, better cosmetic outcomes, and reduced wound complications.	Requires extensive surgical experience; risk of flap necrosis and wound dehiscence.	High patient satisfaction with good cosmetic results and low recurrence rates.
Use of Biological Meshes	Incorporates biologically compatible meshes to reinforce the surgical area and promote tissue integration.	Reduces the risk of recurrence, promotes natural tissue healing, and minimizes complications associated with synthetic materials.	High cost; potential for immune response or rejection in some patients.	Improved long-term outcomes with lower recurrence rates and good integration with native tissue.
Negative Pressure Wound Therapy (NPWT)	Applies controlled negative pressure to the wound site to enhance healing and reduce infection risk.	Accelerates wound healing, reduces edema, decreases bacterial load, and promotes tissue granulation.	Requires continuous device management and regular dressing changes; not suitable for all wound types.	Effective in managing complex or infected wounds, reducing healing time, and improving outcomes.

Comparative effectiveness

Recent studies have underscored the comparative effectiveness of traditional versus advanced surgical techniques, focusing on clinical outcomes, recurrence rates, patient satisfaction, and cost-effectiveness. The choice of surgical method can significantly affect both immediate recovery and long-term health outcomes for patients [25]. When comparing robotic surgery to traditional techniques, several advantages are evident. Robotic surgery is celebrated for its precision and minimally invasive nature, which often results in shorter hospital stays and faster recovery times due to smaller incisions and reduced trauma to surrounding tissues. Clinical studies indicate that patients undergoing robotic procedures generally experience fewer complications than traditional methods [26]. Additionally, robotic surgery is associated with lower recurrence rates, making it a preferred option for complex cases. Similarly, laparoscopic surgery has demonstrated benefits over open surgery, including quicker recovery and reduced postoperative pain. Although open surgery offers greater visibility and control, it is linked with increased tissue damage and longer hospital stays. Laparoscopic techniques, on the other hand, deliver comparable long-term outcomes with lower recurrence rates for various conditions, including colorectal diseases [27]. Patient satisfaction and quality of life are essential factors in evaluating surgical techniques. Research indicates that robotic and laparoscopic surgery patients often report higher satisfaction levels due to reduced postoperative discomfort and a quicker return to daily activities. This improvement in quality of life is a significant consideration for patients and healthcare providers when selecting the appropriate surgical approach [28]. Cost-effectiveness is another crucial factor in the decision-making process. Robotic surgery typically incurs higher costs due to the expenses associated with robotic systems and their maintenance. In contrast, traditional surgical methods are generally more cost-effective, particularly in resource-limited settings [29]. However, the initial higher costs of robotic surgery may be offset by reduced hospital stays and faster recovery, potentially leading to lower overall healthcare costs in the long run. The economic impact of adopting advanced surgical techniques must be carefully weighed against their benefits, as the long-term savings from reduced complications and improved patient outcomes can justify the investment in advanced technologies. This analysis is vital for healthcare systems that optimize resource allocation while enhancing patient care [30].

Postoperative care and management

Proper postoperative care and management are essential for achieving optimal outcomes following surgical treatment for sacrococcygeal pilonidal sinus (SPS). This involves implementing effective wound care protocols, managing potential complications such as infection and recurrence, and establishing a comprehensive long-term follow-up plan [31]. Effective postoperative wound management is critical for promoting healing and preventing complications. Guidelines typically emphasize the importance of appropriate wound dressing based on the surgical technique, whether primary closure, secondary healing, or flap techniques. The initial dressing should be sterile and changed according to the surgeon's instructions [32]. Gentle wound cleansing with saline or antiseptic agents is recommended to remove debris and minimize infection risk. Regular wound inspection is crucial for the early detection of the signs of infection, such as increased redness, swelling, or discharge. The timing of suture removal will depend on the closure method used, with the surgeon providing specific guidance on when this should occur to optimize healing [33]. Despite advancements in surgical techniques, complications such as infection and recurrence can still occur. Early diagnosis and treatment of postoperative infections are essential, and they may involve antibiotics, wound drainage, or, in severe cases, additional surgical intervention. Surgeons should educate patients on recognizing signs of infection and encourage prompt medical attention if symptoms develop. Recurrence is another significant concern, particularly depending on the surgical technique used. Close monitoring for signs of recurrence is crucial, and patients may require further treatment if a recurrence occurs. Regular follow-up appointments are important for the early identification of these issues, allowing for timely intervention [34]. Long-term follow-up is integral to postoperative care, ensuring patients are monitored for potential complications and long-term outcomes. Patients are generally recommended to attend follow-up visits at three, six, and 12 months after surgery, with annual check-ups thereafter. During these visits, a thorough physical examination should be conducted to assess the surgical site and check for any signs of complications or recurrence. Patient education plays a critical role; individuals should be informed about the importance of maintaining good hygiene, avoiding prolonged sitting, and promptly reporting any concerning symptoms. By adhering to these postoperative care and management guidelines, healthcare providers can significantly enhance patient outcomes, minimize complications, and ensure long-term success in treating sacrococcygeal pilonidal sinus [35].

Future directions

The future directions in managing SPS disease are focused on advancing research, integrating emerging technologies, and refining surgical approaches. Current research highlights the need for further studies to better understand recurrence rates and the long-term effectiveness of various surgical techniques. A systematic review has underscored the lack of consensus on the optimal surgical approach for SPS, revealing a significant opportunity for randomized controlled trials to offer clearer guidance on treatment options. Ongoing clinical trials aim to compare the efficacy of minimally invasive techniques versus traditional excision methods, evaluating recurrence rates, postoperative complications, and recovery times, which are critical for improving patient outcomes [31]. Emerging surgical techniques and technologies are being explored to enhance treatment efficacy for SPS. Techniques such as laser therapy, endoscopic approaches, and novel flap designs are gaining attention. Laser-assisted surgery, for example, has shown promise in reducing recovery times and minimizing complications. Additionally, advancements in imaging technologies may improve preoperative planning and intraoperative guidance, potentially leading to better surgical outcomes. The integration of minimally invasive techniques, such as the Karydakis and Limberg flaps, continues to evolve, with ongoing modifications aimed at reducing recurrence rates and enhancing patient comfort [36]. Despite these advancements, several areas require further improvement in SPS management. Standardizing protocols for surgical interventions is essential to minimize variability in outcomes. The high recurrence rates associated with midline closure techniques suggest more effective methods are needed. Enhancing patient education regarding postoperative care and lifestyle modifications could significantly impact recurrence rates. Regular follow-up and monitoring are crucial for the early identification and management of complications [31]. Finally, much-existing literature on SPS management consists of single-centre studies and non-randomized trials. There is a call for more robust, multicenter randomized controlled trials to provide high-quality evidence that can inform clinical practice. In conclusion, the future of SPS management appears promising with ongoing research, the introduction of innovative surgical techniques, and a focus on improving patient care protocols. Addressing identified research gaps and standardizing treatment approaches will enhance outcomes in patients with sacrococcygeal pilonidal sinus disease [37]. Future directions in the treatment of sacrococcygeal pilonidal sinus are summarized in Table 3.

**Table 3:** Future Directions in the Treatment of Sacrococcygeal Pilonidal Sinus (SPS)

Future Directions	Description
Minimally Invasive Techniques	Development and refinement of less invasive procedures to reduce recovery time and minimize scarring.
Improved Wound Healing Strategies	Research on novel wound healing agents and dressings to enhance recovery and reduce complications.
Personalized Treatment Approaches	Tailoring surgical techniques based on patient-specific factors, such as anatomy and disease severity.
Enhanced Imaging and Diagnostic Tools	Use of advanced imaging technologies for better preoperative planning and precise surgical interventions.
Robotic-Assisted Surgery	Exploration of robotic-assisted techniques to improve surgical precision and reduce postoperative pain.
Biologic and Regenerative Therapies	Investigation of biologic materials and regenerative therapies to promote tissue healing and prevent recurrence.
Long-term Outcomes Research	Conducting comprehensive studies to evaluate long-term outcomes and patient satisfaction with new techniques.
Multidisciplinary Care Models	Developing integrated care models involving surgeons, dermatologists, and wound care specialists for comprehensive management.
Patient Education and Compliance	Enhancing patient education to improve compliance with postoperative care and reduce recurrence rates.
Telemedicine and Remote Monitoring	Utilizing telemedicine platforms for follow-up care to monitor wound healing and provide timely interventions.

## Conclusions

In conclusion, the management of sacrococcygeal pilonidal sinus has evolved significantly, with advancements in surgical techniques offering new opportunities to improve patient outcomes. Traditional methods, such as excision with primary closure and flap-based repairs, have provided effective results but are accompanied by varying rates of recurrence and complications. Recent innovations, including minimally invasive techniques and robotic-assisted surgeries, have shown promise in reducing postoperative pain, shortening recovery times, and minimizing recurrence rates. This comprehensive review underscores the importance of selecting the most appropriate surgical approach based on individual patient factors and highlights the need for continued research to refine these techniques further. By integrating the latest advancements and evidence-based practices, clinicians can enhance treatment efficacy and improve the quality of life for patients suffering from sacrococcygeal pilonidal sinus. Future research should focus on comparative studies of new and traditional methods, long-term outcomes, and cost-effectiveness to ensure continued progress in managing this challenging condition.
